# Efficacy of immune checkpoint inhibitors in non-small cell lung cancer with uncommon histology: a propensity-score-matched analysis

**DOI:** 10.1186/s12890-021-01681-6

**Published:** 2021-10-02

**Authors:** Koichi Miyashita, Masato Karayama, Yusuke Inoue, Hironao Hozumi, Yuzo Suzuki, Kazuki Furuhashi, Tomoyuki Fujisawa, Noriyuki Enomoto, Yutaro Nakamura, Masato Kono, Takashi Matsui, Mitsuru Niwa, Keigo Koda, Mikio Toyoshima, Sayomi Matsushima, Shun Matsuura, Kazuhiro Asada, Masato Fujii, Hideki Kusagaya, Hiroyuki Matsuda, Naoki Inui, Takafumi Suda

**Affiliations:** 1 Second Division, Department of Internal Medicine, Hamamatsu University School of Medicine, 1-20-1 Handayama, Hamamatsu, 431-3192 Japan; 2 Department of Chemotherapy, Hamamatsu University School of Medicine, 1-20-1 Handayama, Hamamatsu, 431-3192 Japan; 3 Department of Respiratory Medicine, Seirei Hamamatsu General Hospital, 2-12-12 Sumiyoshi, Hamamatsu, 430-8558 Japan; 4 Department of Respiratory Medicine, Seirei Mikatahara General Hospital, 3453 Mikatahara-cho, Hamamatsu, 433-8558 Japan; 5 Department of Respiratory Medicine, Hamamatsu Medical Center, 328 Tomitsuka-cho, Hamamatsu, 432-8580 Japan; 6 Department of Respiratory Medicine, Hamamatsu Rosai Hospital, 25 Shougen-cho, Hamamatsu, 430-8525 Japan; 7 Department of Respiratory Medicine, Iwata City Hospital, 513-2 Ohkubo, Iwata, 438-8550 Japan; 8 Department of Respiratory Medicine, Fujieda Municipal General Hospital, 4-1-11 Surugadai, Fujieda, 426-8677 Japan; 9 Department of Respiratory Medicine, Shizuoka General Hospital, 4-27-1 Kita-ando, Shizuoka, 420-0881 Japan; 10 Department of Respiratory Medicine, Shizuoka City Hospital, 10-93 Ote-cho, Shizuoka, 420-8630 Japan; 11 Department of Respiratory Medicine, Shizuoka Saiseikai Hospital, 1-1-1 Oshika, Shizuoka, 422- 8527 Japan; 12 Department of Respiratory Medicine, Japanese Red Cross Shizuoka Hospital, 8-2 Otemachi, Shizuoka, 420-0853 Japan; 13 Department of Clinical Pharmacology and Therapeutics, Hamamatsu University School of Medicine, 1-20-1 Handayama, Hamamatsu, 431-3192 Japan

**Keywords:** Pleomorphic carcinoma, Large cell neuroendocrine carcinoma, Not otherwise specified, Programmed death-1, Programmed death ligand-1

## Abstract

**Background:**

Clinical efficacy of immune checkpoint inhibitors (ICIs) for non-small cell lung cancer (NSCLC) with uncommon histology (*u*NSCLC) is unknown.

**Methods:**

Patients with NSCLC treated with ICI monotherapy between January 2014 and December 2018 in 10 Japanese hospitals were retrospectively evaluated. The patients were divided into: (1) NSCLC with common histology (*c*NSCLC), defined as adenocarcinoma and squamous cell carcinoma; and (2) *u*NSCLC, defined as incompatibility with morphological and immunohistochemical criteria for adenocarcinoma or squamous cell carcinoma. Propensity score matching was performed to balance the two groups.

**Results:**

Among a total of 175 patients included, 44 with *u*NSCLC (10 pleomorphic carcinomas, 9 large cell neuroendocrine carcinomas, 2 large cell carcinomas, and 23 not otherwise specified) and 44 with matched *c*NSCLC (32 adenocarcinomas and 12 squamous cell carcinomas) were selected for analyses. Median progression-free survival (PFS) (4.4 months, 95% confidence interval [CI] 1.8–7.7 months) and overall survival (OS) (11.4 months, 95% CI 7.4–27.4 months) in the *u*NSCLC patients were not significantly different from those in matched *c*NSCLC patients (5.4 months, 95% CI 3.1–7.6 months, *p* = 0.761; and 14.1 months, 95% CI 10.6–29.6 months, *p* = 0.381). In multivariate analysis, Eastern Cooperative Oncology Group performance status (ECOG-PS) of 0–1 and programmed death ligand-1 (PD-L1) expression were predictive for PFS and OS in *u*NSCLC.

**Conclusions:**

ICIs had similar clinical efficacy for treatment of *u*NSCLC and *c*NSCLC. Good ECOG-PS and PD-L1 expression were predictive for efficacy of ICIs in *u*NSCLC.

**Supplementary Information:**

The online version contains supplementary material available at 10.1186/s12890-021-01681-6.

## Introduction

The emergence of immune checkpoint inhibitors (ICIs) has led to major changes in treatment paradigms for non-small cell lung cancer (NSCLC). Pembrolizumab, an anti-programmed death-1 (PD-1) antibody, or atezolizumab, an anti-programmed death ligand-1 (PD-L1) antibody, have demonstrated survival benefits over platinum-based chemotherapy in chemo-naïve patients with NSCLC [1, 2]. In previously treated patients with NSCLC, pembrolizumab, atezolizumab and nivolumab (anti-PD-1 antibody) have demonstrated long-term survival benefits over docetaxel [3–6]. Clinical guidelines recommend ICIs as first- second- or later-line treatments for unresectable NSCLC [7–9].

Although adenocarcinoma and squamous cell carcinoma are the dominant tumor pathologies in NSCLC, 8–18% of patients have uncommon histology, such as pleomorphic carcinoma, large cell neuroendocrine carcinoma (LCNEC), large cell carcinoma and not otherwise specified (NOS) [10–13]. As well as distinct histological features, NSCLC with uncommon histology (*u*NSCLC) has different clinical courses and poor therapeutic responses and prognosis compared with NSCLC with common histology (*c*NSCLC), such as adenocarcinoma or squamous cell carcinoma. For example, pleomorphic carcinoma of the lung is reported to progress aggressively and to be refractory to chemotherapy, with an objective response rate (ORR) of 17% and progression-free survival (PFS) of 2 months [14]. Patients with NSCLC-NOS are reported to have a median PFS of 5.9 months after first-line platinum-based chemotherapy, which is shorter than 7.3 months in patients with adenocarcinoma [15]. Patients with LCNEC have better clinical benefit from small cell lung cancer (SCLC)-based chemotherapy, such as etoposide/platinum, compared with NSCLC-based chemotherapy, such as gemcitabine/platinum, pemetrexed/platinum and paclitaxel/platinum [16].

However, little is known about the therapeutic benefits of ICIs for *u*NSCLCs. Some clinical trials for ICIs in NSCLC have included *u*NSCLCs; however, the proportion of *u*NSCLCs in the total study populations was only 2–7% [4, 5, 17, 18]. Given the distinct features and poor therapeutic responses to cytotoxic chemotherapy, it is unknown whether patients with *u*NSCLC have similar clinical benefits from ICIs as those with *c*NSCLC. In this multicenter retrospective study, we compared the efficacy of ICIs in patients with *u*NSCLC or *c*NSCLC using propensity-score-matched analysis. Additionally, we identified predictive factors for ICIs in patients with *u*NSCLC.

## Materials and methods

### Study design

This was a multicenter, retrospective cohort study that was approved by the Institutional Review Board of each participating institution. Patient consent was waved because it was a retrospective study. This study was registered with the University Hospital Medical Information Network (ID: UMIN000037777).

### Patients

We retrospectively reviewed medical records of patients who were diagnosed with advanced or recurrent NSCLC between January 2014 and December 2018 in 10 hospitals in Japan. Patients with pathologically diagnosed NSCLC who received ICI monotherapy were included. Any lines of treatment were allowed if ICI monotherapy was administered. The recurrent stage was defined as recurrence after radical surgery and applicable for systemic therapy, but not for local therapy. Patients who received combination therapy with platinum-based chemotherapy and ICIs or had histories of previous ICI therapy were excluded. The patients were divided into 2 groups on the basis of pathological diagnosis: (1) *c*NSCLC, patients with adenocarcinoma or squamous cell carcinoma; and (2) *u*NSCLC, those without morphological and immunohistochemical criteria for adenocarcinoma or squamous cell carcinoma, such as pleomorphic carcinoma, large cell carcinoma, LCNEC or NOS. Pathological diagnosis was performed morphologically and immunologically at each institution.

### Data collection

Clinical data, including age, sex, smoking history, pathology, PD-L1 tumor proportion score (TPS), cancer staging, Eastern Cooperative Oncology Group performance status (ECOG-PS), line of treatment, and type of ICI were obtained from the patients’ medical records. The responses to ICI were evaluated in accordance with the Response Evaluation Criteria in Solid Tumors (RESIST) version 1.1 [19]. Disease control rate (DCR) was defined as complete response (CR) plus partial response (PR) plus stable disease, and ORR as CR plus PR. PFS and OS were calculated from the date of first administration of ICI.

### Propensity score matching

To balance the baseline of the two groups, 1:1 propensity score matching was performed. Propensity scores were calculated using a logistic regression model and included the following variables: age, sex, smoking status, cancer stage, PD-L1 TPS, line of ICI, and ECOG-PS.

### Statistical analysis

Fisher’s exact test and Mann–Whitney U test were used for categorical and continuous variables, respectively. Kaplan–Meier method and the log-rank tests were used for PFS and OS. Cox proportional hazards regression analysis was used to identify predictive variables for PFS and OS. Logistic regression analysis was used to identify predictive variables for ORR and DCR. Variables of *p* < 0.100 in univariate analyses, pathology (*u*NSCLC vs. *c*NSCLC), and PD-L1 expression were included for multivariate analyses. All values are expressed as median (range) or number (%). A *p* value < 0.05 was considered significant. All statistical analyses were performed with EZR (Saitama Medical Center, Jichi Medical University, Saitama, Japan), which is a graphical user interface for R (The R Foundation for Statistical Computing, Vienna, Austria, version 2.13.0) [20].

## Results

### Patient characteristics

**Fig. 1:**
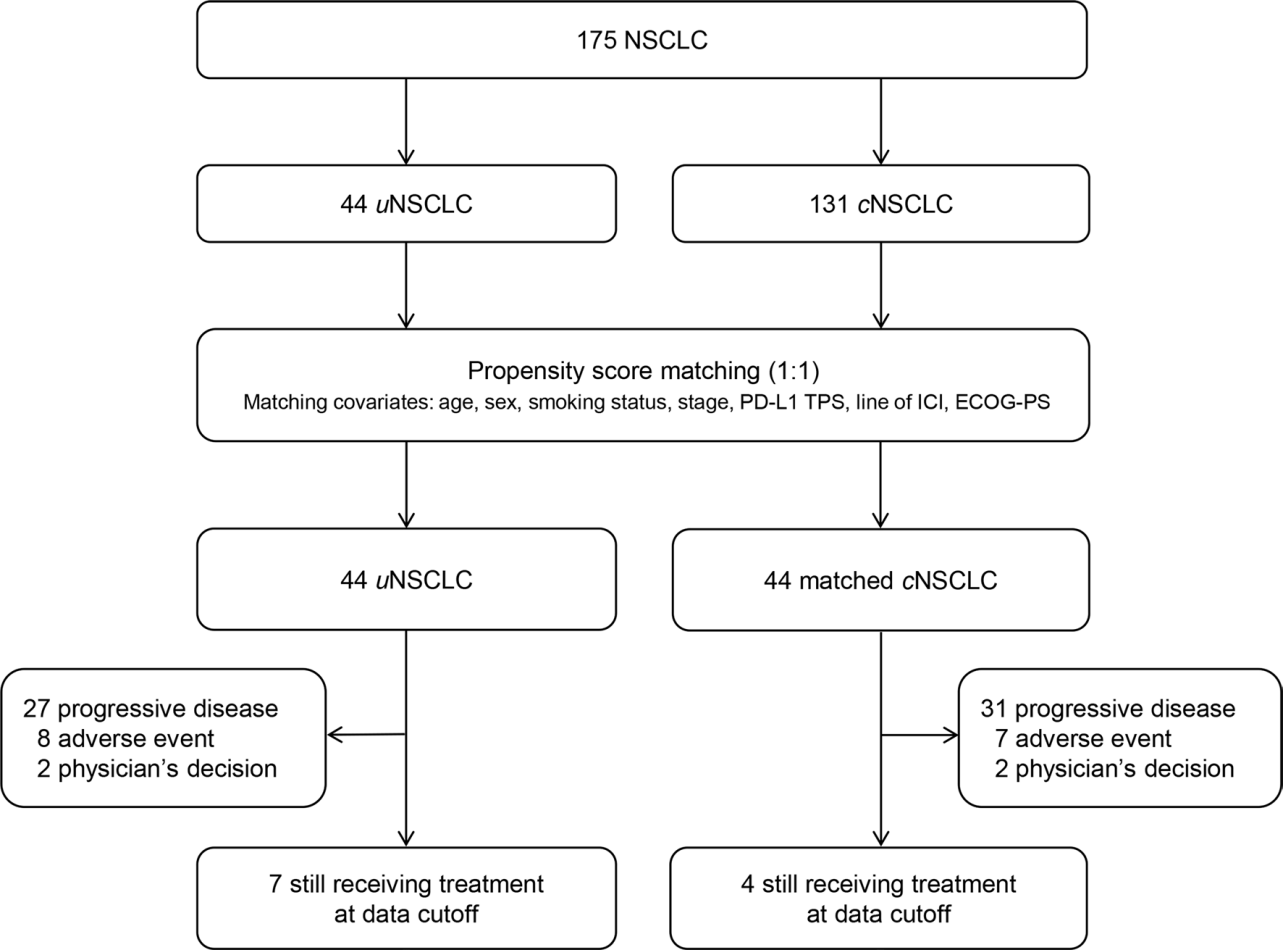
Diagram of study patients. ECOG-PS, Eastern Cooperative Oncology Group performance status; ICI, immune checkpoint inhibitor; NSCLC, non-small cell lung cancer; PD-L1, programmed death ligand-1; TPS, tumor proportion score

A total of 175 patients (44 *u*NSCLCs and 131 *c*NSCLCs) were included in the study. Patient characteristics are shown in Table 1. The *u*NSCLC group had a median age of 66 years, and most patients were men (95%), and most had a smoking history (95%) and good ECOG-PS of 0–1 (82%). Eight (18%), 10 (23%), and 16 (36%) patients in the *u*NSCLC group had brain, liver and bone metastases, respectively. The histological types were 10 (23%) pleomorphic carcinomas, 9 (20%) LCNECs, 2 (5%) large cell carcinomas, and 23 (52%) NOSs. No patient had *epidermal growth factor receptor* (*EGFR*) mutation or *anaplastic lymphoma kinase* (*ALK*) fusion. The expression of tumor PD-L1 was TPS ≥ 50% in 17 (39%) patients, 1–49% in 10 (23%), < 1% in 6 (14%), and not available in 11 (25%). The ICIs were administered as first-, second- or later-line in 7 (16%), 22 (50%) and 15 (34%) patients, respectively. Of these, 22 (50%), 16 (36%) and 6 (14%) patients received nivolumab, pembrolizumab and atezolizumab, respectively. The unmatched *c*NSCLC group had a significantly lower proportion of men (*p* = 0.010), higher proportion of stage IV disease (*p* = 0.004), better ECOG-PS (*p* = 0.001), and less liver metastasis (*p* = 0.039) compared with the *u*NSCLC group. A significantly higher proportion of patients in the unmatched *c*NSCLC group was not evaluated the tumor PD-L1 status (*p* < 0.001). Seven (5%) patients and one (1%) patient in the unmatched *c*NSCLC group had *EGFR* gene mutation and *ALK* fusion gene, respectively. After 1:1 propensity score matching, 44 patients with *c*NSCLC were selected (matched *c*NSCLC) (Fig. 1). The matched *c*NSCLC group had comparable patients’ demographics to the *u*NSCLC group. Only one patient had an active driver mutation (in *EGFR*) in the matched *c*NSCLC group.

**Table 1:** Patient characteristics

	Unmatched *c*NSCLC	Matched *c*NSCLC	*u*NSCLC	*p*-value
	(n = 131)	(n = 44)	(n = 44)	
Age, years	69 (43–83)	67 (44–81)	66 (40–83)	0.923
Sex, men	103 (79)	42 (95)	42 (95)	1.000
Smoking status, ever-smokers	111 (85)	42 (95)	42 (95)	1.000
EGOG-PS,				0.237
0	69 (53)	21 (48)	14 (32)	
58 (44)	19 (43)	22 (50)		
≥ 2	4 (3)	4 (9)	8 (18)	
Stage,				0.715
III	23 (18)	14 (32)	14 (32)	
IV	97 (74)	24 (55)	21 (48)	
Recurrence	11 (8)	6 (14)	9 (20)	
Metastases,				
Brain	26 (20)	14 (32)	8 (18)	0.218
Liver	13 (10)	4 (9)	10 (23)	0.143
Bone	30 (23)	11 (25)	16 (36)	0.355
Pathology,				< 0.001
Adenocarcinoma	85 (65)	32 (73)	0 (0)	
Squamous cell carcinoma	46 (35)	12 (27)	0 (0)	
Pleomorphic carcinoma	0 (0)	0 (0)	10 (23)	
LCNEC	0 (0)	0 (0)	9 (20)	
Large cell carcinoma	0 (0)	0 (0)	2 (5)	
Not otherwise specified	0 (0)	0 (0)	23 (52)	
PD-L1: TPS,				0.942
≥ 50%	20 (15)	15 (34)	17 (39)	
1–49%	20 (15)	12 (27)	10 (23)	
< 1%	16 (12)	5 (11)	6 (14)	
NA	75 (57)	12 (27)	11 (25)	
Line of treatments,				0.209
1st	7 (5)	6 (14)	7 (16)	
2nd	61 (47)	15 (34)	22 (50)	
≥ 3rd	63 (48)	23 (52)	15 (34)	
Treatments,				< 0.001
Nivolumab	123 (94)	37 (84)	22 (50)	
Pembrolizumab	8 (6)	7 (16)	16 (36)	
Atezolizumab	0 (0)	0 (0)	6 (14)	

The data are expressed as number (%) and median (range)

*c*NSCLC, common non-small cell lung cancer; ECOG-PS, Eastern Cooperative Oncology Group performance status; LCNEC, large cell neuroendocrine carcinoma; NA, not available; PD-L1, programmed death ligand-1; TPS, tumor proportion score; *u*NSCLC, uncommon non-small cell lung cancer

In the *u*NSCLC group, 7 (16%) patients were still receiving ICIs at the time of data cutoff and the remaining 37 (84%) were not receiving ICIs because of progressive diseases (n = 27), adverse events (n = 8) or physician’s decision (n = 2) (Fig. 1). In the matched *c*NSCLC group, only 4 (9%) patients were still receiving ICIs while the other 40 (91%) were not receiving ICIs because of progressive diseases (n = 31), adverse events (n = 7) or physician’s decision (n = 2). The median follow-up time was 11.9 months (range 0.1–43.8 months).

### Efficacy of ICIs

**Fig. 2:**
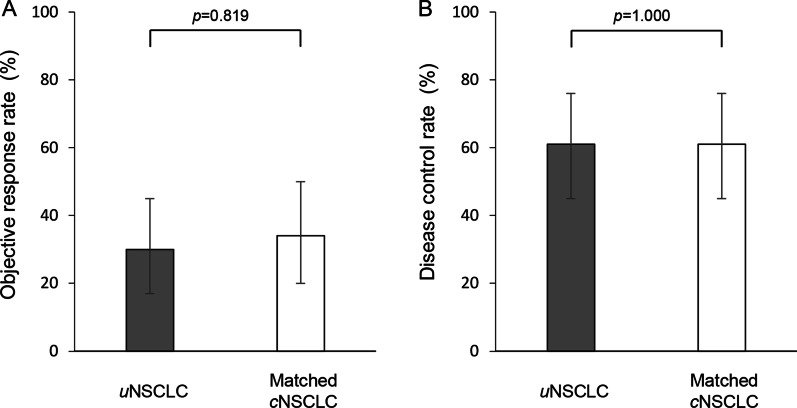
Objective response rate (ORR) and disease control rate (DCR). (A) ORR and (B) DCR in uncommon non-small cell lung cancer (*u*NSCLC, gray bar) and propensity-score-matched common NSCLC (*c*NSCLC, white bar). Error bars indicate 95% confidence interval

**Fig. 3:**
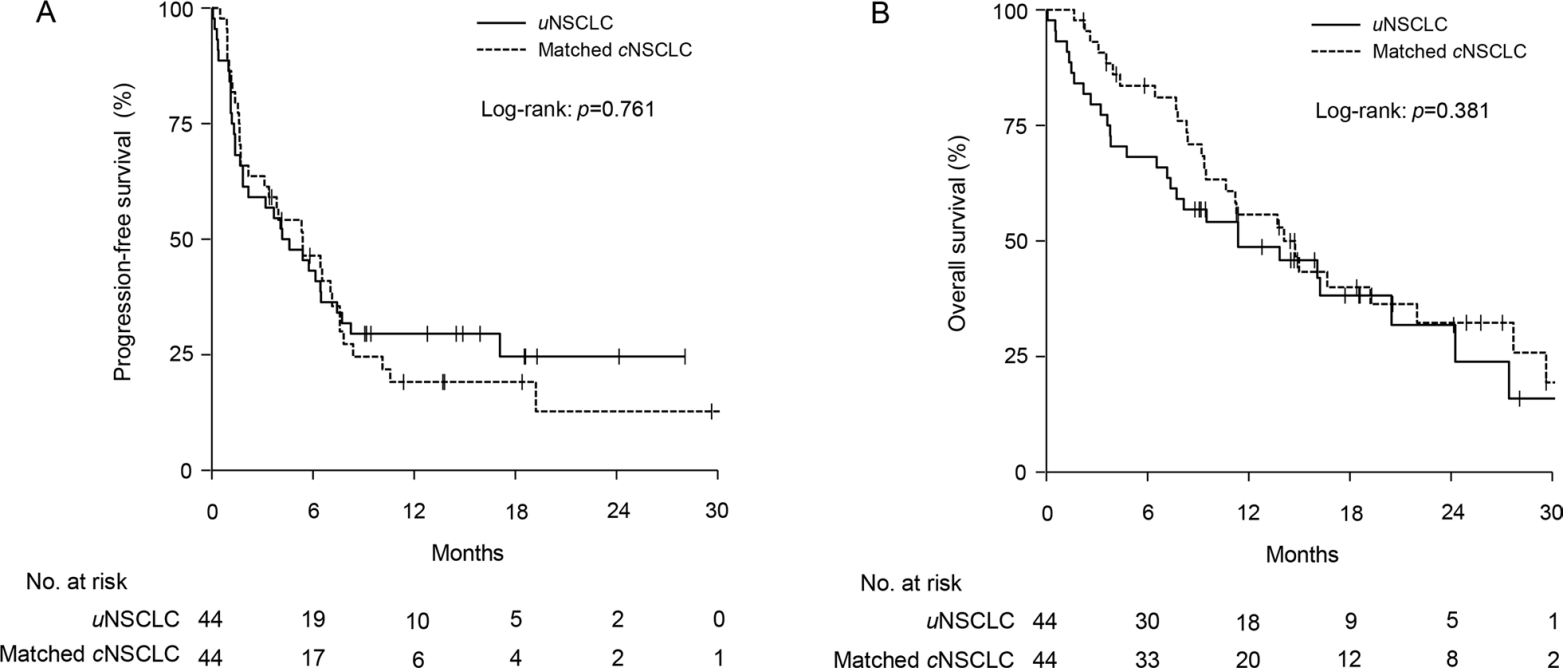
Kaplan–Meier curves for progression-free survival (PFS) and overall survival (OS). **A** PFS and **B** OS in uncommon non-small cell lung cancer (*u*NSCLC, solid line) and propensity-score-matched common NSCLC (*c*NSCLC, dashed line)

The ORR of 30% (95% confidence interval [CI], 17–45%) in the *u*NSCLC group was comparable with 34% (95% CI, 20–50%) in the matched *c*NSCLC group (*p* = 0.819) (Fig. 2A) (Table 2). The DCR of 61% (95% CI, 45–76%) in the *u*NSCLC group was comparable with 61% (95% CI, 45–76%) in the matched *c*NSCLC group (*p* = 1.000) (Fig. 2B) (Table 2). The median PFS of 4.4 months (95% CI 1.8–7.7 months) in the *u*NSCLC group was not significantly different from 5.4 months (95% CI 3.1–7.6 months) in the matched *c*NSCLC group (*p* = 0.761) (Fig. 3A). The median OS of 11.4 months (95% CI 7.4–27.4 months) in the *u*NSCLC group was comparable with 14.1 months (95% CI 10.6–29.6 months) in the matched *c*NSCLC group (*p* = 0.381) (Fig. 3B).

**Table 2:** Overall response

	Unmatched *c*NSCLC	Matched *c*NSCLC	*u*NSCLC	*p*-value
	**(n = 131)**	**(n = 44)**	**(n = 44)**	
Response,				0.492
CR	0 (0)	0 (0)	2 (5)	
PR	37 (28)	15 (34)	11 (25)	
SD	31 (24)	12 (27)	14 (32)	
PD	63 (48)	17 (39)	17 (39)	
ORR	28 (21–37)	34 (20–50)	30 (17–45)	0.819
DCR	52 (43–61)	61 (45–76)	61 (45–76)	1.000

The data are expressed as number (%) and rate (95% confidence interval)

*c*NSCLC, common non-small cell lung cancer; CR, complete response; DCR, disease control rate; ORR, objective response rate; PD, progressive disease; PR, partial response; SD, stable disease; uNSCLC, uncommon non-small cell lung cancer

### Univariate and multivariate analyses for efficacy of ICIs

In univariate analysis, PD-L1 expression and first-line treatment were predictive for ORR, and ECOG-PS and PD-L1 expression were predictive for DCR (Additional file 1: Tables S1 and S2). In multivariate analysis, PD-L1 expression was predictive for ORR and DCR, and ECOG-PS was predictive for ORR (Additional file 1: Tables S1 and S2). In univariate Cox proportional hazard analysis, ever-smokers, ECOG-PS of 0–1, and PD-L1 expression (both TPS ≥ 50% and ≥ 1%) were significant predictive factors for PFS. In multivariate analysis, ECOG-PS of 0–1 and PD-L1 expression were independent predictive factors for PFS (Table 3). Ever-smokers and ECOG-PS of 0–1 were independent predictive factors for OS in multivariate analysis, while PD-L1 TPS ≥ 50% demonstrated a borderline predictive significance and TPS ≥ 1% did not (Table 4). Meanwhile, *u*NSCLC was not predictive for ORR, DCR, PFS or OS. Additionally, the presence of brain, liver, and bone metastases or line of ICI treatment was not predictive for ORR, DCR, PFS or OS. When limited to the patients with *u*NSCLC, ECOG-PS of 0–1 and PD-L1 expression were independent predictive factors for PFS and OS (Additional file 1: Tables S3 and S4).

**Table 3:** Cox proportional hazard analysis for progression-free survival

	Univariate		Multivariate			
			Set 1		Set 2	
	h (95%CI)	*p*-value	HR (95%CI)	*P*-value	HR (95%CI)	*p*-value
Age, ≥ 65	1.13 (0.68–1.87)	0.645				
Sex, men	1.00 (0.31–3.19)	0.996				
Smoking status, ever-smokers	0.35 (0.13–1.00)	0.049	0.51 (0.17–1.54)	0.232	0.35 (0.12–1.08)	0.068
ECOG-PS, 0–1	0.37 (0.19–0.71)	0.003	0.42 (0.21–0.84)	0.015	0.45 (0.23–0.89)	0.022
Stage, III	0.96 (0.57–1.62)	0.889				
Pathology, *u*NSCLC (vs. *c*NSCLC)	0.93 (0.57–1.50)	0.757	1.00 (0.61–1.63)	0.996	1.01 (0.62–1.65)	0.970
PD-L1,						
≥ 50%	0.43 (0.25–0.75)	0.003	0.44 (0.26–0.77)	0.004		
≥ 1%	0.58 (0.36–0.95)	0.029			0.56 (0.33–0.94)	0.029
Line of treatment, 1st-line	0.81 (0.39–1.71)	0.584				

CI, confidence interval; *c*NSCLC, common non-small cell lung cancer; ECOG-PS, Eastern Cooperative Oncology Group performance status; HR, hazard ratio; PD-L1, programmed death ligand-1; *u*NSCLC, uncommon non-small cell lung cancer

**Table 4:** Cox proportional hazard analysis for overall survival

	Univariate		Multivariate			
			Set A		Set B	
	HR (95%CI)	*p*-value	HR (95%CI)	*P*-value	HR (95%CI)	*p*-value
Age, ≥ 65	1.16 (0.67–2.00)	0.606				
Sex, men	0.80 (0.25–2.57)	0.704				
Smoking status, ever-smokers	0.25 (0.09–0.71)	0.009	0.30 (0.10–0.90)	0.032	0.28 (0.09–0.84)	0.023
ECOG-PS, 0–1	0.34 (0.16–0.71)	0.004	0.38 (0.18–0.82)	0.014	0.39 (0.19–0.84)	0.015
Stage, III	0.73 (0.41–1.32)	0.298				
Pathology, *u*NSCLC (vs. *c*NSCLC)	1.27 (0.75–2.16)	0.381	1.29 (0.75–2.22)	0.352	1.25 (0.72–2.16)	0.434
PD-L1,						
≥ 50%	0.61 (0.34–1.10)	0.102	0.58 (0.32–1.06)	0.076		
≥ 1%	0.88 (0.51–1.51)	0.638			0.80 (0.45–1.42)	0.451
Line of treatment, 1st-line	1.10 (0.49–2.46)	0.812				

CI, confidence interval; *c*NSCLC, common non-small cell lung cancer; ECOG-PS, Eastern Cooperative Oncology Group performance status; HR, hazard ratio; PD-L1, programmed death ligand-1; *u*NSCLC, uncommon non-small cell lung cancer

### Subgroup analyses of histological subtypes

Patients with *u*NSCLC were evaluated on the basis of histological subtype (Additional file 1: Table S5). In pleomorphic carcinoma, tumor PD-L1 showed TPS ≥ 50% in 5 (50%) patients, 1–49% in 3 (30%) patients, and < 1% in 0 (0%) patients; PD-L1 was not evaluated in 2 patients (20%). In LCNEC, TPS was ≥ 50% in 1 (11%) patient, 1–49% in 1 (11%) patient, < 1% in 4 (44%) patients, and not evaluated in 3 (33%) patients. In NOS, TPS was ≥ 50% in 9 (39%) patients, 1–49% in 6 (26%) patients, < 1% in 2 (9%) patients, and not evaluated in 6 (26%). The median PFS and OS were 7.7 months (95% CI: 0.4 months–not estimated [NE]) and 9.5 months (95% CI: 1.2 months–NE) in pleomorphic carcinoma, respectively; 1.3 months (95% CI: 0.1 months–NE) and 3.8 months (95% CI: 0.1 months–NE) in LCNEC; and 4.1 months (95% CI: 1.8–6.4 months) and 13.8 months (95% CI: 6.5–24.2 months) in NOS (Additional file 1: Fig. S1 A–B). No significant difference in PFS and OS was observed in subtypes in the *u*NSCLC group compared with the matched *c*NSCLC group.

Patients in the matched *c*NSCLC were also evaluated separately on the basis of their histological subtypes (Additional file 1: Table S6). The expression of tumor PD-L1 was TPS ≥ 50% in 13 (41%) patients, 1–49% in 8 (25%), < 1% in 3 (9%), and not evaluated in 8 (25%) in adenocarcinoma; and ≥ 50% in 2 (17%), 1–49% in 4 (33%), < 1% in 2 (17%), and not evaluated in 4 (33%) in squamous cell carcinoma. The median PFS and OS were 4.2 months (95% CI: 1.8–7.8 months) and 16.8 months (95% CI: 13.7–29.6 months) in adenocarcinoma; and 3.2 months (95% CI: 1.8–5.3 months) and 12.5 months (95% CI: 9.2–19.4 months) in squamous cell carcinoma (Additional file 1: Fig. S2 A-B). There was no significant difference in PFS (*p* = 0.132) and OS (*p* = 0.070) between adenocarcinoma and squamous cell carcinoma.

## Discussion

In the current study, we found that ICIs were efficacious for patients with *u*NSCLC and those with *c*NSCLC with comparable demographic characteristics after propensity score matching. Good ECOG-PS and high PD-L1 expression were significant predictive factors for efficacy of ICIs, regardless of tumor histology. Patients with *u*NSCLC are known to demonstrate insufficient response to chemotherapy. However, our data indicate that ICIs may provide therapeutic benefits even for patients with *u*NSCLC, especially those who have good ECOG-PS and high PD-L1 expression.

The median PFS of 4.4 months and median OS of 11.4 months after ICI monotherapy in the current study were comparable with those in previous studies of ICI monotherapy in patients who mostly had *c*NSCLC (PFS, 2.3–4.0 months and OS, 9.2–13.8 months) [3–6]. In a retrospective study of 21 patients with LCNEC who received ICI monotherapy, median PFS and OS were 4.2 and 11.8 months, respectively [21]. In 49 patients with pulmonary pleomorphic carcinoma who received ICI monotherapy, median PFS and OS were 7.2 and 22.2 months, respectively [22]. Given that conventional chemotherapies for NSCLC often provide limited survival benefits for lung cancer with uncommon histology, ICI monotherapy can be considered as a treatment option [14–16].

Tumor PD-L1 expression is a gold standard biomarker for the efficacy of ICIs in NSCLC; however, the level of tumor PD-L1 expression and its predictive ability varies among different tumor types. For example, only 13.5% of patients with gastric cancer had PD-L1 TPS ≥ 1% and the efficacy of nivolumab was not associated with PD-L1 expression [23]. Furthermore, in renal cell carcinoma, 11% and 24% of patients had PD-L1 TPS ≥ 5% and ≥ 1%, respectively, and the efficacy of nivolumab was not associated with PD-L1 expression [24]. Although *u*NSCLC has different pathological features from *c*NCSLC, tumor PD-L1 expression (TPS ≥ 1%) was observed in ~ 60% of the patients with *u*NSCLC and was also predictive for efficacy of ICIs.

Good ECOG-PS, a well-known predictive factor for the efficacy of ICIs in NSCLC, was also predictive in *u*NSCLC [11, 12, 25, 26]. Although precise mechanisms underlying ECOG-PS and the efficacy of ICIs are unknown, poor general condition may reflect deteriorated host immune status and lead to weakened effector T cells. When compared with *c*NSCLC, *u*NSCLC tends to progress rapidly and be resistant to standard chemotherapy [14, 15]. Therefore, it is suggested that patients with *u*NSCLC are predisposed toward poor general condition without adequate treatments. Approximately 20% of the patients with *u*NSCLC had poor ECOG-PS ≥ 2, compared with only 3% of those with unmatched *c*NSCLC. Our data suggest that early initiation of ICIs may be considered for patients with *u*NSCLC, especially if they have high PD-L1 expression and good ECOG-PS.

There were two main limitations to this study. First, differences in PD-L1 expression and the efficacy of ICIs among different histological subtypes of *u*NSCLC were unknown, because of the limited number of patients and 25% of the patients did not undergo PD-L1 testing. It is reported that 80% of patients with pleomorphic carcinoma had high PD-L1 expression and favorable clinical response to ICIs (median PFS 7.2 months and median OS 22.2 months) [22]. Only 10–22% of patients with LCNEC had PD-L1 expression and had median PFS of 4.2 months and median OS of 11.8 months [21, 27, 28]. In the current study, the patients with pleomorphic carcinoma had the highest proportion of PD-L1 expression and the longest PFS, whereas those with LCNEC had the lowest PD-L1 and the worst PFS. It is possible that the clinical impact of PD-L1 expression and efficacy of ICIs differed owing to the histological subtypes of *u*NSCLC. Second, we only evaluated ICI monotherapy. Several single or combination therapeutic strategies for ICIs have emerged, such as cytotoxic T-lymphocyte antigen-4 antibody therapy, combination therapy with ICI and chemotherapy, and combinations of different ICI agents [17, 18, 29]. The clinical benefits of the novel ICIs for *u*NSCLC are unknown and should be investigated further.

## Conclusions

ICIs had similar clinical efficacy for treatment of *u*NSCLC and *c*NSCLC. Additionally, good ECOG-PS and high PD-L1 expression were predictive for the efficacy of ICIs in *u*NSCLC.

## Supplementary Information

**Additional file 1**.** Table S1**. Logistic regression analysis for objective response.** Table S2**. Logistic regression analysis for disease control rate.** Table S3**. Cox proportional hazard analysis for progression-free survival in uncommon non-small cell lung cancer.** Table S4**. Cox proportional hazard analysis for overall survival in uncommon non-small cell lung cancer.** Table S5**. Patient characteristics according to histological subtypes in uncommon non-small cell lung cancer.** Table S6**. Patient characteristics according to histological subtypes in matched common non-small cell lung cancer.** Fig. S1**. Kaplan-Meier curves for progression-free survival and overall survival by histology in uncommon non-small cell lung cancer group.** Fig. S2**. Kaplan-Meier curves for progression-free survival and overall survival by histology in matched common non-small cell lung cancer group.

## Data Availability

The datasets used and/or analyzed during the current study are available from the corresponding author on reasonable request.
