# Supplementary material for: Efficacy of immune checkpoint inhibitors in non-small cell lung cancer with uncommon histology: a propensity-score-matched analysis
Source: BMC Pulm Med. 2021 Oct 2;21:309. doi: 10.1186/s12890-021-01681-6 (PMC8487118; doi:10.1186/s12890-021-01681-6)

Additional file

Table S1. Logistic regression analysis for objective response

	Univariate		Multivariate			
			Set 1		Set 2	
	OR (95%CI)	*p*-value	OR (95%CI)	*p*-value	OR (95%CI)	*p*-value
Age, ≥65	0.53 (0.21–1.34)	0.183				
Sex, men	1.42 (0.14–14.30)	0.765				
ECOG-PS, 0–1	6.06 (0.74–49.47)	0.093	6.18 (0.68–56.22)	0.106	5.07 (0.57–45.08)	0.146
Stage, III	0.35 (0.12–1.05)	0.061	0.42 (0.13–1.42)	0.165	0.45 (0.13–1.52)	0.198
Pathology, *u*NSCLC (vs. *c*NSCLC)	0.81 (0.33–1.99)	0.647	0.85 (0.30–2.45)	0.764	0.89 (0.32–2.49)	0.825
PD-L1, TPS						
≥50%	7.63 (2.80–20.81)	<0.001	6.12 (1.82–20.63)	0.003		
≥1%	8.91 (2.43–32.69)	<0.001			5.52 (1.39–22.00)	0.015
Line of treatment, 1st-line	4.40 (1.29–15.04)	0.018	1.17 (0.25–5.38)	0.842	2.30 (0.60–8.79)	0.224

CI, confidence interval; *c*NSCLC, common non-small cell lung cancer; ECOG-PS, Eastern Cooperative Oncology Group performance status; OR, odds ratio; PD-L1, programmed death ligand-1; TPS, tumor proportion score; *u*NSCLC, uncommon non-small cell lung cancer

Table S2. Logistic regression analysis for disease control rate

	Univariate		Multivariate			
			Set 1		Set 2	
	OR (95%CI)	*p*-value	OR (95%CI)	*p*-value	OR (95%CI)	*p*-value
Age, ≥65	1.14 (0.47–2.77)	0.772				
Sex, men	1.63 (0.22–12.11)	0.636				
ECOG-PS, 0–1	6.12 (1.52–24.61)	0.011	6.21 (1.40–27.57)	0.016	6.00 (1.41–25.55)	0.015
Stage, III	1.20 (0.47–3.04)	0.701				
Pathology, *u*NSCLC (vs. *c*NSCLC)	1.00 (0.42–2.36)	1.000	1.12 (0.43–2.90)	0.823	1.16 (0.46–2.93)	0.755
PD-L1, TPS						
≥50%	4.33 (1.55–12.15)	0.005	3.77 (1.05–13.55)	0.042		
≥1%	1.78 (0.74–4.28)	0.200			1.18 (0.45–3.10)	0.741
Line of treatment, 1st-line	4.09 (0.85–19.76)	0.079	1.40 (0.20–9.69)	0.733	3.63 (0.67–19.59)	0.134

CI, confidence interval; *c*NSCLC, common non-small cell lung cancer; ECOG-PS, Eastern Cooperative Oncology Group performance status; OR, odds ratio; PD-L1, programmed death ligand-1; TPS, tumor proportion score; *u*NSCLC, uncommon non-small cell lung cancer

12 **Table S3. Cox proportional hazard analysis for progression-free survival in uncommon non-small cell lung cancer**

	Univariate		Multivariate			
			Set 1		Set 2	
	HR (95%CI)	*p*-value	HR (95%CI)	*p*-value	HR (95%CI)	*p*-value
Age, ≥65	1.16 (0.57–2.35)	0.680				
Sex, men	2.12 (0.29–15.61)	0.460				
Smoking status, ever-smokers	0.43 (0.10–1.84)	0.256				
ECOG-PS, 0–1	0.31 (0.13–0.74)	0.008	0.23 (0.09–0.59)	0.002	0.27 (0.11–0.66)	0.004
Stage, III	0.53 (0.24–1.18)	0.121				
PD-L1: TPS,						
≥50%	0.53 (0.25–1.12)	0.097	0.42 (0.19–0.93)	0.032		
≥1%	0.46 (0.22–0.93)	0.032			0.41 (0.20–0.86)	0.017
Line of treatment, 1st-line	1.01 (0.39–2.64)	0.981				

CI, confidence interval; ECOG-PS, Eastern Cooperative Oncology Group performance status; HR, hazard ratio; PD-L1, programmed death ligand-1

Table S4. Cox proportional hazard analysis for overall survival in uncommon non-small cell lung cancer

	Univariate		Multivariate			
			Set 1		Set 2	
	HR (95%CI)	*p*-value	HR (95%CI)	*p*-value	HR (95%CI)	*p*-value
Age, ≥65	1.96 (0.85–4.50)	0.112				
Sex, men	2.50 (0.33–18.99)	0.375				
Smoking status, ever-smokers	0.39 (0.09–1.68)	0.206				
ECOG-PS, 0–1	0.36 (0.14–0.90)	0.029	0.30 (0.12–0.78)	0.014	0.35 (0.14–0.88)	0.026
Stage, III	0.51 (0.20–1.26)	0.143				
PD-L1,						
≥50%	0.53 (0.23–1.20)	0.129	0.46 (0.20–1.07)	0.072		
≥1%	0.47 (0.22–1.01)	0.053			0.46 (0.21–0.99)	0.048
Line of treatment, 1st-line	0.96 (0.33–2.80)	0.939				

CI, confidence interval; ECOG-PS, Eastern Cooperative Oncology Group performance status; HR, hazard ratio; PD-L1, programmed death ligand-1

Table S5. Patient characteristics according to histological subtypes in uncommon non-small cell lung cancer

	Pleomorphic carcinoma (n=10)	LCNEC (n=9)	Large cell carcinoma (n=2)	NOS (n=23)
Age, years	67 (53–80)	70 (61–82)	62 (58–66)	65 (40–83)
Sex, men	9 (90)	9 (100)	2 (100)	22 (96)
Smoking status, ever-smokers	8 (80)	9 (100)	2 (100)	23 (100)
ECOG-PS,				
0	0 (0)	3 (33)	1 (50)	10 (43)
1	7 (70)	3 (33)	1 (50)	11 (48)
≥2	3 (30)	3 (33)	0 (0)	2 (9)
Stage,				
III	2 (20)	2 (22)	0 (0)	10 (44)
IV	3 (30)	5 (56)	0 (0)	13 (57)
Recurrence	5 (50)	2 (22)	2 (100)	0 (0)

Metastases,				
Brain	0 (0)	0 (0)	0 (0)	8 (35)
Liver	5 (50)	4 (44)	0 (0)	1 (4)
Bone	5 (50)	3 (33)	2 (100)	6 (26)
PD-L1: TPS,				
≥50%	5 (50)	1 (11)	2 (100)	9 (39)
1–49%	3 (30)	1 (11)	0 (0)	6 (26)
<1%	0 (0)	4 (44)	0 (0)	2 (9)
NA	2 (20)	3 (33)	0 (0)	6 (26)
Line of treatments,				
1st	0 (0)	0 (0)	1 (50)	6 (26)
2nd	7 (70)	4 (44)	1 (50)	10 (44)
≥3rd	3 (30)	5 (56)	0 (0)	7 (30)
Treatments,				
Nivolumab	6 (60)	5 (56)	0 (0)	11 (48)
Pembrolizumab	4 (40)	1 (11)	2 (100)	9 (39)

Atezolizumab	0 (0)	3 (33)	0 (0)	3 (13)

The data are expressed as number (%) and median (range).

ECOG-PS, Eastern Cooperative Oncology Group performance status; LCNEC, large cell neuroendocrine carcinoma; NA, not available; NOS, not otherwise specified; PD-L1, programmed death ligand-1; TPS, tumor proportion score

Table S6. Patient characteristics according to histological subtypes in matched common non-small cell lung cancer

	Adenocarcinoma (n=32)	Squamous cell carcinoma (n=12)
Age, years	66 (60–70)	70 (66–75)
Sex, men	30 (94)	12 (100)
Smoking status, ever-smokers	30 (94)	12 (100)
ECOG-PS,		
0	18 (56)	3 (25)
1	11 (34)	8 (67)
≥2	3 (9)	1 (8)
Stage,		
III	8 (25)	6 (50)
IV	20 (62)	4 (33)
Recurrence	4 (12)	2 (17)
Metastases,		

Brain	13 (41)	1 (8)
Liver	4 (12)	0 (0)
Bone	10 (31)	1 (8)
PD-L1: TPS,		
≥50%	13 (41)	2 (17)
1–49%	8 (25)	4 (33)
<1%	3 (9)	2 (17)
NA	8 (25)	4 (33)
Line of treatments,		
1st	5 (16)	1 (8)
2nd	10 (31)	5 (42)
≥3rd	17 (53)	6 (50)
Treatments,		
Nivolumab	26 (81)	11 (92)
Pembrolizumab	6 (19)	1 (8)
Atezolizumab	0 (0)	0 (0)

The data are expressed as number (%) and median (range).

ECOG-PS, Eastern Cooperative Oncology Group performance status; NA, not available; PD-L1, programmed death ligand-1; TPS, tumor proportion score

Fig. S1. Kaplan-Meier curves for progression-free survival and overall survival by histology in uncommon non-small cell lung cancer group

(A)Progression-free survival and (B)overall survival in pleomorphic carcinoma (solid line), large cell neuroendocrine carcinoma (LCNEC, dashed line) and not otherwise specified (NOS, dotted line).

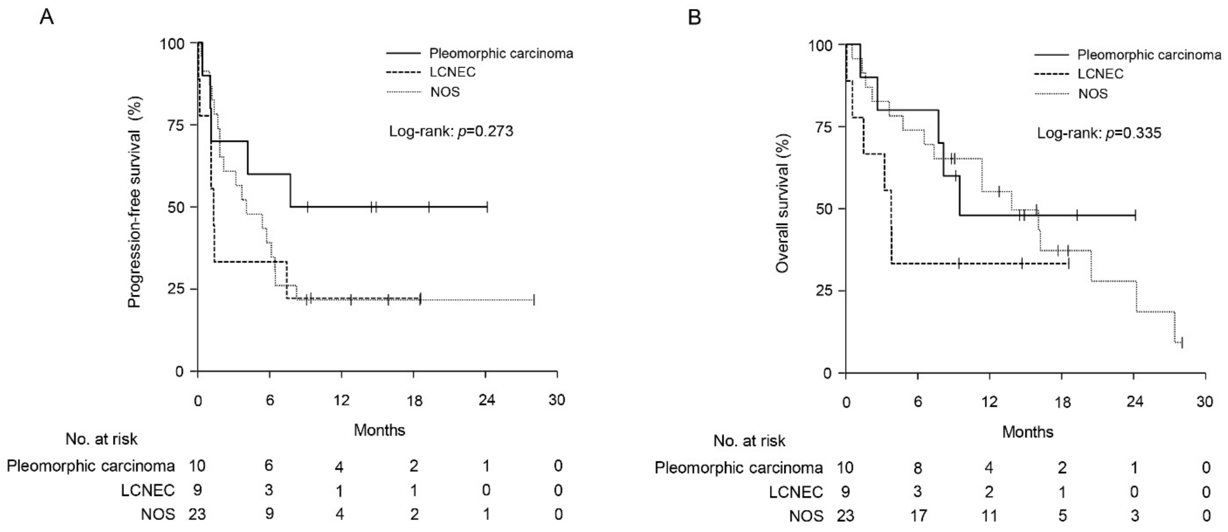

Fig. S2. Kaplan-Meier curves for progression-free survival and overall survival by histology in matched common non-small cell lung cancer group

(A)Progression-free survival and (B)overall survival in adenocarcinoma (solid line) and squamous cell carcinoma (dashed line).

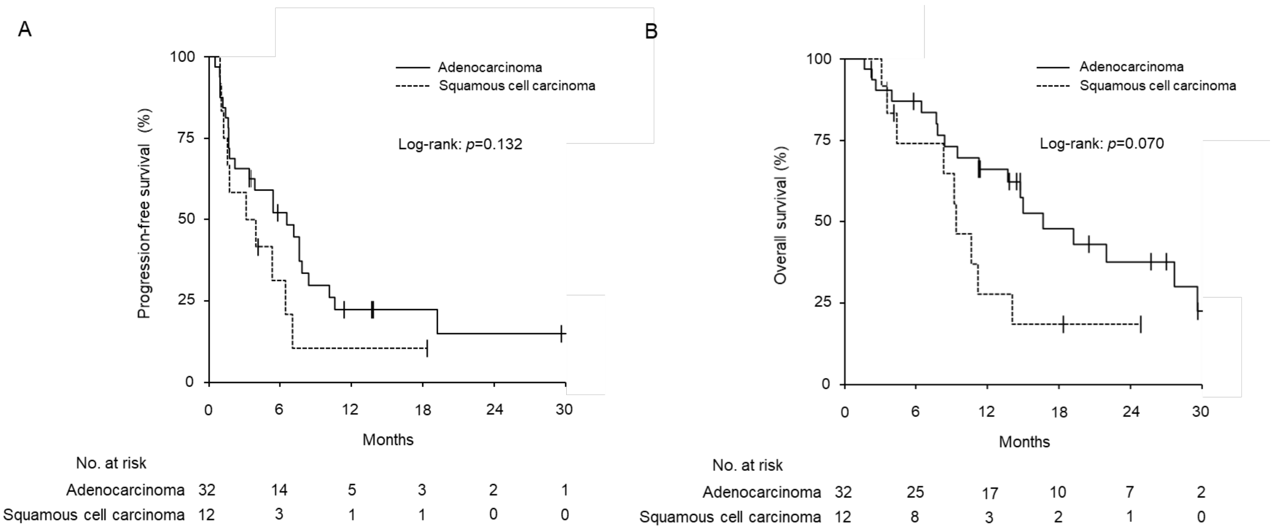

Supplement: Supplementary file 1 — Additional file 1. Table S1. Logistic regression analysis for objective response. Table S2. Logistic regression analysis for disease control rate. Table S3. Cox proportional hazard analysis for progression-free survival in uncommon non-small cell lung cancer. Table S4. Cox proportional hazard analysis for overall survival in uncommon non-small cell lung cancer. Table S5. Patient characteristics according to histological subtypes in uncommon non-small cell lung cancer. Table S6. Patient characteristics according to histological subtypes in matched common non-small cell lung cancer. Fig. S1. Kaplan-Meier curves for progression-free survival and overall survival by histology in uncommon non-small cell lung cancer group. Fig. S2. Kaplan-Meier curves for progression-free survival and overall survival by histology in matched common non-small cell lung cancer group. [file 12890_2021_1681_MOESM1_ESM.pdf]
